# The Path Is Made by Walking—Mapping the Healthcare Pathways of Parents Continuing Pregnancy after a Severe Life-Limiting Fetal Diagnosis: A Qualitative Interview Study

**DOI:** 10.3390/children9101555

**Published:** 2022-10-13

**Authors:** Kerstin Hein, Franziska Flaig, Annika Schramm, Gian Domenico Borasio, Monika Führer

**Affiliations:** 1 Center for Pediatric Palliative Care, University Hospital, LMU Munich, 81377 Munich, Germany; 2 Palliative and Supportive Care Service, Lausanne University Hospital and University of Lausanne, 1011 Lausanne, Switzerland

**Keywords:** palliative care, perinatal care, pediatrics, prenatal diagnosis, bereavement, qualitative research

## Abstract

In Germany, research on experiences and care pathways of parents continuing pregnancy after a life-limiting fetal diagnosis is scarce. There are several recommendations but few structured programs. We aimed to explore experiences and needs of parents, reconstruct their care pathways, and identify requirements for a perinatal palliative care program. We conducted semi-structured interviews with 11 mothers and 9 fathers and analyzed data using the Saldaña’s Coding Method. Codes were organized in templates to reconstruct care pathways. Pathways started with a suspicious finding prompting a referral to prenatal diagnostics. Parents experienced severe emotional distress during prenatal diagnostics due to scarce information, insensitiveness, and a perceived pressure towards abortion. As a result, they overlooked referrals to psychosocial counseling, generating a care gap. Most parents reached the decision to continue pregnancy without professional support. They then chose a trusted midwife or gynecologist as main caregiver during pregnancy. There were no regular referrals to palliative care, which mainly became relevant when the child survived. Our data indicate that a perinatal palliative care program requires early and comprehensive information, sensitivity, and a non-directive approach. Already existing support services need to be identified and connected through structured pathways, with a particular focus on midwives.

## 1. Introduction

The increased use of prenatal diagnostics has led to a growing number of diagnoses of life-limiting fetal conditions [1,2,3]. Learning about a severe fetal condition is devastating and traumatic [4,5,6,7,8,9,10]. Parents react with shock, distress, and feelings of disbelief [11]. They read the verbal and non-verbal cues from the sonographer and become alert when the interaction changes [6,9]. They gain the impression that providers are insensitive [11], and do not disclose all options. Many of them feel pushed towards abortion [4,7,9,12,13].

Parents continuing pregnancy believe in the value of life [11,13,14,15]. Many of them feel judged because of their decision [13] and fear substandard care [9,12]. They recognize their baby as a person [9,14,15,16,17,18,19], call him or her by name [15,17,18,19], and expect providers to show respect [12,13,14,19,20,21]. Parents hope to meet their child alive [14,15,22] and try to find meaning in his or her brief life [15,17,18]. They want to be recognized and treated as parents [7]. After perinatal loss, families feel urged to bid farewell and feel regret if they are not able to do so [23]. Memory making helps them to create evidence and supports their bereavement [24,25].

Perinatal palliative care supports families who choose to continue pregnancy following a life-limiting fetal diagnosis [26,27,28,29,30]. It addresses the perinatal care of pregnant women and the neonatal care of infants [27,29]. Perinatal palliative care has been described as a viable alternative to abortion for families who choose this path [28,30,31,32].

In Germany, mothers who receive the diagnosis of a serious fetal condition are entitled to obtain cost-free medical and psychosocial counseling [8,33,34]. In most cases, medical counseling is provided by prenatal diagnostic physicians [2,8]. Physicians must inform pregnant women about the option of psychosocial counseling [2,8,10,33,34].

After the diagnosis, parents require routine antenatal care and anticipatory planning to make complex decisions [4,30,35,36], to anticipate possible outcomes and care options [2,31,37] and to develop goals of care for the prenatal, perinatal and neonatal period [2,4,32]. It includes the development of a birth plan with medical and non-medical goals [2,3,4,8,28,30,31,32,35,37,38,39,40,41,42].

Perinatal palliative care includes end-of-life care, follow-up after hospital discharge, and bereavement support [3,32,35,38]. In Germany, mothers are entitled to postnatal care provided by midwives. Families may also apply for special nursing services [8,43] or household support [8].

There has been increasing interest and a growing number of publications with recommendations for perinatal and neonatal palliative care [3,41,44]. However, clinical studies are rare [45] and the evidence supporting the existing perinatal palliative care programs is still scarce [46]. Most programs have been developed in the USA [44,47] while European perinatal palliative care structures remain poorly developed [1,45]. The only known structured perinatal palliative care program in Germany is located at the Center for Perinatal Medicine at the Charité in Berlin [48].

The aim of this study is to enhance our knowledge about care experiences and needs of parents who decide to continue pregnancy despite the life-limiting condition of their unborn child and to reconstruct their pathway through existing healthcare structures.

Parental care experiences can be seen as subjective interpretations of their encounters with care providers [49,50]. The sequence of care experiences related to the management of a particular health condition across all touchpoints with the healthcare system corresponds to a healthcare pathway, also known as patient journey. Healthcare pathways are always defined from the point of view of patients [49,50,51,52]. Mapping healthcare pathways means to reconstruct the entire end-to-end healthcare process [52], assess care experiences and needs of patients [52,53], describe problems and gaps [51,52,54], and suggest improvements to the care process [54].

By reconstructing parental healthcare pathways, we expect to identify requirements for the development of a structured perinatal palliative care programme at the Center for Pediatric Palliative Care in Munich.

## 2. Materials and Methods

### 2.1. Study Design

Mapping healthcare pathways allows the use of diverse methodological approaches and techniques [52,54,55]. As healthcare pathways correspond to a subjective interpretation of care experiences, we deemed it best to use a qualitative methodological approach to reconstruct parental care experiences across the healthcare system.

Qualitative research seeks to explore and understand the meanings individuals or groups assign to their experiences. The focus lies on the description of subjective concepts rather than on explaining the causes of a particular phenomenon. Qualitative researchers are interested in the characteristics and complexity of real life contexts and single cases [56,57]. Samples tend to be small and non-representative [58]. Qualitative research does not seek generalization but transferability of results, which does not depend on the number but on the type of participants involved in the study [59,60,61]. The aim of qualitative studies is to explore and identify relevant topics rather than to quantify them. The purpose is to develop data-driven hypotheses rather than verifying them [57].

The method and its reporting follow the Standards for Reporting Qualitative Research (SRQR) [62].

### 2.2. Sampling and Recruitment

We purposively selected bereaved mothers and fathers who continued pregnancy following a severe and life-limiting fetal diagnosis, which means that the fetus was at a high risk to die in utero, during delivery, or in the first years of life. Even though we aimed at including couples, we accepted parents whose partners declined participation. According to Tseng [63] and Hughes [64], bereavement is most intense during the first six up to twelve months after perinatal loss. Afterwards, grief levels and severity of symptoms fall over time [63,64,65]. Therefore, we only included couples whose child died at least one year previously. Exclusion criteria was insufficient German language skills.

Participants were contacted through gatekeepers with access to a network of midwives across Bavaria. We also approached families who consulted the pediatric palliative care team at the Center for Pediatric Palliative Care of the LMU University Hospital in Munich between 2005 and 2013 after learning about a life-limiting diagnosis for their unborn child.

### 2.3. Data Collection

A psychologist (A.S.) and a physician and midwife (F.F.) conducted semi-structured, narrative-oriented interviews with parents between November 2017 and January 2020. Mothers and fathers were interviewed separately at their domicile. Interview topics included parental care experiences starting with pregnancy, followed by diagnosis, decision-making, the time around birth and death, and afterwards. Parents were able to recall vivid details about their experience and needed little input to tell their stories. The average duration of the interviews was 76 min (Range: 45–111 min). Interviews were recorded on audio tape, transcribed verbatim, and pseudonymized. Audio files and transcripts were stored on institutional, password-protected servers. Only the research team had access to data.

### 2.4. Data Analysis

We analyzed data between March 2018 and September 2020 by means of the Coding Method of Saldaña [66], which organizes the analysis in cycles of coding. During first cycle coding, A.S. and a psychologist specialized in qualitative research methods (K.H.) independently read the transcripts to detect patterns and generate codes using a combination of structural, descriptive, in vivo, and evaluative coding. During second cycle coding, codes were clustered and merged into broader categories. Categories were compared with each other, refined, and organized into a storyline following the sequence of events. Memos were written during the whole process. Analysis was supported by MAXQDA 12. The resulting codes were validated in multidisciplinary research meetings and used to compile templates. The compilation of templates allowed us to reconstruct the particular healthcare pathway of each family.

## 3. Results

We approached 12 families. One of them was not interested in participation as they had moved to another region. Eleven families agreed to participate in the study. Six of these families were recruited through gatekeepers. Five families had approached the pediatric palliative care team in Munich in the past. In nine cases, we conducted separate interviews with both parents. In two families, we only met mothers as fathers declined participation without further explanation. Most parents had completed a high educational level and were either Catholics or Protestants. A detailed characterization of the sample can be found in Table 1.

Although the average time passed since the diagnosis was 5 years (Range: 2–10 years), almost all participants could vividly recall the experience.

### 3.1. Prenatal Diagnosis

Healthcare pathways started with a suspicious finding during routine antenatal ultrasonography controls prompting a referral to specialized prenatal diagnostics. Parents described prenatal diagnostics as the absolute low point of the whole experience. Mothers as well as fathers became anxious when the interaction with the sonographer changed during the examination. Some physicians stopped communicating with parents; others described with excessive detail all defects of the fetus. Participants recalled this experience as insensitive, and disrespectful.

*It [Prenatal diagnostics] was lousy. What we experienced was absolutely and completely unacceptable. This was the worst of medical art. First she [prenatal diagnostician] says, she [the baby] has an incurable disease and will only survive a few days, if at all. Already five sentences later she says: We can remove her and then you try again. It was completely pathetic (…) It was the beginning of the whole story and at the same time the negative highlight. (Father 8)*

The bad news only partially explain the emotional distress. Parents also missed sufficient and meaningful information, a better guidance through existing structures of care, and neutral advice. Parents had the impression that diagnosticians were one-sided and pushed them towards abortion.

*The situation was so bizarre. She [prenatal diagnostician] just left. She should have stayed. She should have told us, I see something suspicious. I want to take the time to explain this, and to provide information to help us understand, offer us a framework. (Mother 11)*

*We would have wished them to disclose all our options. They only said: ‘Most parents in your situation decide to have an abortion’. They did not say: ‘You can carry the pregnancy to term’ and what we have to face afterwards, nobody told us. (Mother 2)*

Several physicians were unavailable for further consultations. Only few offered parents a separate room to collect themselves after the ultrasound. Subsequently, most parents felt lost and displaced.

*You leave the examination room. You stay in the corridor. In front of the registration desk. At some point, they return you the maternity logbook. You could cry or you compose yourself. People either look at you or ignore you. As if you weren’t there. You don’t know what to do with yourself. You cannot entrench yourself forever in the toilet. You are completely confused. Yet you have to make it outside and see how you manage to continue. (Mother 1).*

*They told us the diagnosis and sent us home. They should have had something there (…) a room, to collect yourself, where you can recapitulate and think. What did just happen? (Father 11)*

### 3.2. The Care Gap: What Now?

Most prenatal diagnosticians offered verbal or written contact details of psychosocial services. Yet participants were in a state of shock and overlooked this information, which created a care gap. Couples who were referred to human genetics for further clarification felt disappointed. They interpreted the information as redundant and unrelated to their everyday life.

*Before that we had an appointment with a human geneticist (…) This was not really instructive or helpful, I would say. She drew some kind of family trees of our parents and grandparents, how this is inherited. However, we already knew this because we did our own research after the diagnosis. (Father 5)*

Most parents did not know whom to turn to after the ultrasound. They felt disoriented, helpless, and abandoned.

*I left the place surrounded by a fog. I knew my child is not doing well. But I was feeling all right. She was moving. My feelings were actually good. I left the place completely confused. I did not know what was happening. Then I called my husband and cried. I said: ‚I do not know anything. My child. Either she is severely disabled or she is doing fine.’ It seems like a mix-up. Honestly, I did not know anything anymore. (Mother 1)*

Disorientation resulted from insufficient understanding of the diagnosis and its practical consequences. Almost all participants searched the internet, consulted books and specialized journals. However, particularly the internet proved to be more confusing than helpful. Parents missed a contact person to collect and guide them through the process. Most sought advice from an already known and trusted gynecologist or midwife.

*However, in the end, this was the diagnosis and we were standing on the street, confronted with this thing, and thought, what now? I think, what we would have needed acutely, in the first moment, was somebody to collect us. Somebody to answer the first questions that spontaneously crossed our minds. (Father 11)*

### 3.3. Decision-Making: Let My Child Decide

Almost all participants reached the decision to continue with pregnancy without professional support. This happened despite the fact that during a normal pregnancy in Germany regular visits by a gynecologist are scheduled. For many mothers, the desire to keep the baby resulted from the contradiction they felt during the ultrasound between the announcement of the diagnosis and their own perception of fetal movements.

*This was my baby. She was in my belly. She was kicking. She was happy (…) and it was like: Whoa! This would be murder (…) I cannot do this. I do not want to. I will not. (Mother 4)*

Fathers on the other hand were concerned about the consequences of having a severely sick or disabled child. Their first impulse was to protect the already existing family.

*I think, whatever happens, even an unborn child does not have the right to blow up a whole family (…) Just imagine, a handicapped child is born and the family falls apart (…) This would be very bad. (Father 7)*

In most cases, a common decision was reached within a few days. Couples organized a timeout to reflect the situation. Herein, fathers resolved their skepticism and supported the decision of mothers. Difficulties in finding a common ground caused emotional distress and challenged the integrity of couples. Finally, all participants agreed that life is a gift and that the baby should decide whether to live or not.

*If the child wants to die, it will die on its own. I am not the one who has the right to decide whether the child will live or die (…) I will not do this. Let her decide on her own. (Mother 1)*

### 3.4. Parental Needs and Care Pathways during Pregnancy and Birth

The remaining pregnancy was associated with ambivalent feelings. Respondents wanted to bond and spend time with the baby. They named their child and organized activities to collect memories. Yet they also felt burdened by anticipatory grief and constantly worried whether the baby was still alive. Religious or spiritual beliefs helped them to make sense of the situation.

Parental accounts revealed the existence of several well-functioning healthcare services. However, care provision was scattered. There were no structured pathways, no coordinating institution, and consequently no continuous care. Participants had to make their own path by walking. Table 2 informs about the access to existing structures of care as described by parents during the interviews.

Participants appreciated care providers who offered them customized solutions. They also valued when care providers accepted their choices and showed respect towards the baby.

During pregnancy, parents attended routine examinations performed by gynecologists and midwives who became the main contact persons and the principal touchpoints with the healthcare system during this period of time. Participants were particularly fond of midwives. Midwives guided parents through pregnancy and the healthcare system, prepared them for upcoming events, performed rituals, and offered birth and bereavement support.

Participants visited several hospitals before choosing one. At the hospital, they had access to a multidisciplinary team, with whom they had at least one appointment for anticipatory planning. However, many parents associated the hospital setting with anonymous care and avoided frequent contacts with the hospital team.

Some care providers referred parents to pediatric palliative care services. However, this was not a regular offer. Palliative care services also provided anticipatory guidance, but played an overall minor role during the prenatal phase.

Other forms of support included psychosocial counselling via private networks and housekeeping and childcare services requested from the health insurance.

Table 3 shows the assessment of care providers as expressed by parents during the interviews.

Four babies died in utero between the 11th and 20th week of gestation. Three died during or shortly after birth. Two newborns died during their first week of life while two other children survived four and seven months, respectively.

All but two deliveries took place at the hospital. All mothers described giving birth as a beautiful experience, regardless of the survival of the newborn. The majority baptized their child shortly after birth. Participants appreciated the sensitive and personalized care provided by the hospital staff.

### 3.5. Parenting: Spending Time with the Child

Parents whose child survived, cherished the short time they spent with their living offspring. They collected memories and celebrated each day.

*Then, we just celebrated her birthday each day (…) There was cake and ice cream every day. (Mother 8)*

At the same time, parents were constantly alert due to the perceived imminence of death. They felt alarmed with strange noises and the beeping of machines. Anxiety was reinforced by the difficulty of recognizing the time of death.

*Of course, we had several crises (…) Twice we thought, he was dead. And everybody else thought it too. But then, he came back again. You could tell, he was fighting for his life. (Father 9)*

Three out of four families whose child survived were able to take their child home. This was possible due to good preparation, trusting and accessible contact persons, help with medical care, and domestic support. The main care provider in these cases was the pediatric palliative care team. Participants appreciated their experience, competence, reliability, accessibility, and cooperation with other care providers like pediatricians in private practice.

### 3.6. Farewell Rituals

All participants highlighted farewell rituals as the high point of their experience. Parents spent as much time as possible with their deceased children, collected mementos, shared the experience with relatives and friends, performed rituals, and made creative funeral arrangements. Farewell rituals were essential to comprehend and accept death.

*I did not expect it, but it was important for us to have her home, because you could clearly see how the soul leaves the body. Well, you have seen the child alive before and you have seen it dead one day later, which is not very pleasant, to put it politely. However, as we saw the body and could sense and see and feel how the soul leaves the body, that nobody is home any more in that little body. Seeing this in the course of these days allowed the own soul, the own spirit and mind, the own heart to come along, to comprehend that she has died. (Father 8)*

When children died at the hospital, the staff usually allowed parents to say goodbye in private. Participants were encouraged to hold the deceased newborn. They took pictures, bathed or anointed the baby, made imprints of hands and feet, or wrapped the baby in blankets. One mother was able to hold but not to look at her child and regretted this afterwards. Other parents regretted not having taken enough photographs. Participants took the child to their room or went for a walk with the baby in a basket. Family and friends came to visit the infant.

*Our children came (…) And we went for a walk. I had a basket (…) There we put her and covered her. Then we went with our three children to a playground near the hospital. We made some pictures, how they played. And I was sitting on a rocker with the basket beside me and thought, what would people say if they knew there is a dead baby in the basket. Crazy, right? But this is family life, right? (Mother 7)*

In Bavaria, it is possible to lay out a corpse up to four days at home. This means that parents can take their deceased child home provided a funeral undertaker transports the body [67,68]. All but two families seized this option and unanimously described this as the highlight of their experience. Families were capable of drastic actions when hospitals did not release the body.

*I wrapped her [deceased daughter] up and we went directly home with her, one and a half hours. Exactly. We just held her and went home in our car (…) The only thing was that the hospital administration called us the next day and told us the baby was missing. They said they would sent us the police, because we are not allowed to do this. And we said, okay, we will lock the door. The police will not enter and take away our dead child (Mother 6)*

Families celebrated their deceased child at home. The baby was incorporated in everyday life and accompanied family members during meals or whilst sitting in the living room. Everybody touched, talked, and sang to the baby.

*The burial was on Wednesday. We had her at home until then. We had her in a dark and cool room and got her out once a day in the evenings. We took her to the living room and spent time with her. We sat with her, held her, sang to her. We took pictures. Family pictures on the coach with a self-timer (Mother 7)*

Parents wished to organize the funeral and were unsatisfied when the undertaker did not allow them to do so. Families engaged siblings, relatives, and friends in burial preparations. Most funerals were arranged as birthday parties with colorful balloons and songs for children.

### 3.7. Bereavement

After the burial, parents organized a timeout for grief and remembrance. They felt supported by other bereaved parents, whom they contacted through online forums or support groups. The experience taught them to appreciate what they have, set new priorities in life, become more relaxed and less upset by daily problems. Parents came closer together. None of them had regrets.

Several participants reported a lack of understanding from relatives and friends. About half of the families reported signs of emotional disturbance in siblings such as sudden worsening of school performance, somatic symptoms, emotional breakdown, aggressive behavior, fear of death, and resistance towards the presence of the deceased child at home.

*It left marks on everybody. Our oldest daughter became vegetarian. She was 13 years old and said, she will never have children on her own. She doubted her faith because of what God puts us through (…) Our next daughter had a breakdown after three years. Her subconscious had been dealing with death the whole time (…) She was afraid to go to bed one day and not wake up the next day (…) My next daughter suddenly had back pain, back pain, back pain (…) One of my sons became very aggressive (…) The youngest had a (…) severe peanut allergy, which is life-threatening (…) now he knows, death is definitive. So he stopped eating at school. He refused snacks at school, because he was afraid. (Mother 10)*

Several participants attended special postnatal exercise classes for bereaved parents. Classes helped them to process the experience and meet other bereaved parents. In addition, most mothers and some siblings attended psychosocial counseling. Narratives typically ended with a new pregnancy.

## 4. Discussion

### 4.1. Main Findings

Respondents described fragmented structures of care with already existing but scattered support services. Care coordination was mainly managed by the parents themselves. Parents had to create their own care pathway by walking. This is consistent with a German study on pregnancy counselors who point out that support for such situations is available but uncoordinated [1]. Discontinuous care has also been observed in other countries [16,69,70] with negative effects such as uncertainty, colliding and overlapping services [71], unclear crossover of professional roles, perception of “ownership” of patients [69], increased risk of care gaps, late referrals, conflicting information, miscommunication, or missing documentation [71].

Mapping the healthcare pathways helped us to realize the entire care experience of participants, to identify main touchpoints and relevant care gaps across the healthcare system [51] as well as to understand how families perceive and move between dispersed services [53,54]. Such findings may support the future integration of services into a coordinated and more effective and continuous process of care [51,54].

The starting point and most burdensome part of the healthcare pathways was prenatal diagnostics and counseling. Participants felt disturbed by a perceived pressure towards abortion. This is consistent with the assessment of German pregnancy counselors who think that physicians do not always disclose the option of carrying pregnancy to term [1]. German sonographers are gynecologists and obstetricians who attend certified courses on sonography [72]. They usually have little experience with life-limiting conditions and limited knowledge about neonatal palliative care. They think that continuing pregnancy would be unbearable and recommend abortion [2]. Yet parents would prefer neutral and non-directive counselling [10] with a full disclosure of all available options [73].

It is recommended that prenatal diagnosticians summon a multi-professional team to inform parents about the diagnosis [2,38,39,42]. Unfortunately, most parents did not have access to a multi-professional team at the time of diagnosis. Moreover, parents ignored referrals to pregnancy counseling, which led to a care gap following prenatal testing. From the viewpoint of pregnancy counselors, physicians often disregard psychosocial aspects and omit referrals to counseling [1]. However, since 2010 prenatal diagnosticians must inform and refer parents to psychosocial counseling [8,33,34]. Our findings suggest that diagnosticians comply with these regulations while parents overlook this information. Thus, merely informing and handing out an address seems to be insufficient to motivate couples to use these services [8]. Parents still prefer to seek advice from relatives and friends [10]. Consequently, they reach their decisions without professional support.

The care gap following diagnosis drove respondents to seek a main contact person on their own. Several families chose a midwife as sole care provider or together with a gynecologist. German midwives offer comprehensive physical and psychological care for the mother and the child during pregnancy and the perinatal period, at the hospital or at home [8]. They help families to navigate the healthcare system [1] and provide follow-up care after birth [8]. Specially trained midwives also offer bereavement support [74] and special postnatal exercises [8]. Thus, midwives are in a key position to provide continuous support [1,8,70].

There were no regular referrals to pediatric palliative care services. Palliative care played only a minor role during the prenatal phase. It is conceivable that the stigma associated with palliative care delayed referrals to palliative care services [75]. However, studies suggest that parents appreciate palliative care consultations and wish them to happen sooner [9,16]. Our findings indicate that palliative care became relevant when babies survived. In this case, the palliative care team became the main contact. It provided accessible and continuous care by collaborating with other care providers.

A surprising finding was the extended need to interact with the deceased child. Parents not only collected mementos, talked, touched, or bathed the dead baby. Most of them took the newborn home and recreated a normal family life. Between death and burial, parents still see a child and not a corpse. They need this time to realize and accept death [76,77]. Postmortem rituals offer a sense of control that assists in overcoming helplessness [23,74,76]. Memory making creates tangible evidence and legitimizes parental loss [19,24,25]. Qualitative studies suggest that taking care of a dead baby is a valuable experience for parents [24,77,78,79,80,81]. It reinforces bonding [8,74,76], prevents regrets, supports bereavement, and facilitates recovery [8,64,74,76,82]. Accordingly, it has been incorporated into guidelines and protocols [8,64,78,82]. However, there are studies that question the benefits of holding a dead infant. They argue that evidence is scarce and of poor quality [64,65,81,82] and claim that seeing and holding a dead child may have adverse effects on the mental health and wellbeing of mothers [82,83] and their partners [84]. These findings are in conflict with published qualitative evidence [81,83] and our own findings.

Importantly, we found signs of emotional distress among siblings. Chances are that the focus on the ill or deceased child compromised the capacity of parents to attend sibling grief [85,86]. Siblings may have missed age-appropriate communication and adequate guidance during the process [85,87,88]. Since our data show that siblings may be at risk for a later development of mental health problems, parental counselling for sibling support should be routinely provided in the situation of perinatal death.

### 4.2. Strengths and Weaknesses

To the best of our knowledge, this is the first German study that explores experiences, needs, and care pathways of parents continuing with pregnancy after a life-limiting prenatal diagnosis.

Parents continuing pregnancy are the target population of perinatal palliative care, but still represent a small and exceptional group of families. Almost all expressed religious beliefs, had access to higher education, and substantial social resources. We cannot assume that all affected parents have these resources to cope with the situation. Findings are further based on retrospective accounts. In some cases, several years had passed since the experience. Remembrance may not match actual experience, and it is possible that parents left care providers unmentioned, even though they consulted them in the past.

This was a qualitative study, which means that the sample was small and non-representative. Accordingly, it is not possible to generalize these results. Further research is needed to validate our findings.

## 5. Conclusions

Our data suggest that caring for parents continuing pregnancy after the diagnosis of a life-limiting fetal condition requires comprehensive information, sensitiveness, and a non-directive, personalized approach, especially in the context of prenatal diagnostics. The findings seem to indicate that a structured perinatal palliative care program needs to bridge the care gap after prenatal testing, improve referrals to psychosocial counseling, and reinforce inter-institutional collaboration. The development of such a program should recognize already existing and well-functioning care structures and integrate them through structured pathways. There appears to be a definite need for a multi-professional and inter-institutional network including resources for case management and coordination, which could be provided by pediatric palliative care services in collaboration with midwives.

## Figures and Tables

**Table 1:** Demographic characteristics of participants (*n* = 20).

Characteristics	Mothers	Fathers
**Age (years)** Mean Age Range	42 32–52	46 30–63
**Highest Educational Level** Postgraduate Level University (of Applied Sciences) Vocational School	0 8 3	2 6 1
**Denomination** Catholic Protestant Buddhist Undenominational	5 3 1 2	6 1 0 2
**Family Status at Diagnosis** Married Unmarried Partnership	8 3	
**Diagnosis of the child** Trisomy 18 Anencephaly Turner syndrome with congenital heart defect Encephalocele with congenital heart defect Cytomegalovirus infection with severe CNS and organ manifestations	5 2 2 1 1	
**Time of death of the child** Intrauterine Death Stillbirth Neonatal death (3 days, 7 days) Death during infancy (4 months, 7 months)	4 3 2 2	

Text in bold refers to the type of characteristics.

**Table 2:** Utilization of existing structures of care (*n* = 11).

Phase	Care Structures	Families Mentioning Use of Service
Prenatal Diagnosis	Gynecologist Prenatal Diagnostician Human Geneticist	11 11 3
Decision-Making	Midwife Psychosocial Support Spiritual Support Gynecologist	3 2 2 1
Pregnancy	Midwife Gynecologist Hospital (diverse specialties) Pediatric Palliative Care Team Household Support Psychosocial Support Pediatrician Prenatal Diagnostician Spiritual Support Undertaker Bereavement support Alternative Medicine	9 8 7 5 4 3 3 3 2 2 1 1
Birth	Hospital (diverse specialties) Midwife Spiritual Support Household support Pediatrician	9 5 2 1 1
Spending time with living child	Hospital (diverse specialties) Pediatric Palliative Care Team Nursing services Household support Pediatrician Midwife Spiritual Support	4 3 2 2 1 1 1
Farewell	Undertaker Hospital (diverse specialties) Midwife Spiritual support Bereavement support Pediatric Palliative Care Team Household support	10 5 3 3 1 1 1
Follow up/Bereavement	Midwife Psychosocial support Bereavement support Alternative Medicine Spiritual support	3 3 3 2 1

**Table 3:** Parental assessment of care providers.

Care Provider	What Was Helpful?	What Was Unhelpful?
Gynecologist	Continuity of care—contact person Parent-centred approach Comprehensive Information Sensitiveness	Directive approach—Paternalism Provider seems overburdened
Prenatal Diagnostician		Insensitiveness Directive approach—Paternalism Insufficient, inadequate information
Human Geneticist		Directive approach—Paternalism Insufficient, inadequate information Unnecessary Information
Hospital (diverse specialties)	Comprehensive Information Parent-centred approach Anticipatory guidance and planning Allows privacy, separate room Sensitiveness Helpful referrals Allows participation of siblings Allows visitors	Anonymity—no regular contact person Members of the staff seem overburdened
Midwife	Continuity of care—contact person Parent-centred approach Sensitiveness Comprehensive information Competent, experienced Self-determination, creative design of rituals Anticipatory guidance and planning	
Psychosocial support	Competent, experienced Supports decision-making Supports siblings Supports self-care Anticipatory guidance and planning Supports partnership	Inadequate when unplanned consult on physician’s request
Spiritual support	Continuity of care—contact person Self-determination, creative design of rituals Helpful referrals Competent, experienced	Inadequate when unplanned consult on physician’s request
Bereavement support	Competent, experienced Self-determination, creative design of rituals Supports siblings	
Pediatric Palliative Care Team	Continuity of care—contact person when child survives Anticipatory guidance and planning Comprehensive information Parent-centred approach Sensitiveness Enables going home Helpful referrals Competent, experienced Collaboration with other care providers Support dealing with bureaucracy Allows participation of siblings	
Pediatrician	Continuity of care—contact person when child survives Collaboration with other care providers Anticipatory guidance and planning Parent-centred approach	
Nursing services	Supports medical care of the child Enables going home	
Undertaker	Parent-centred approach Self-determination, creative design of rituals	Directive approach—Paternalism No creative design of rituals
Alternative medicine	Supports self-care	
Household support	Practical help household Support childcare	

## Data Availability

Ethical considerations and data protection protocols do not allow sharing original data with third parties. The interview guideline, the coding list, and the templates are available on demand.
